# Sex-Related Differences in On-Treatment Platelet Reactivity in Patients with Acute Coronary Syndrome

**DOI:** 10.3390/biomedicines13092068

**Published:** 2025-08-25

**Authors:** David Mutschlechner, Anastasios Tsarouchas, Maximilian Tscharre, Patricia Pia Wadowski, Silvia Lee, Joseph Pultar, Constantin Weikert, Simon Panzer, Thomas Gremmel

**Affiliations:** 1 Department of Internal Medicine I, Cardiology and Intensive Care Medicine, Landesklinikum Mistelbach-Gänserndorf, 2130 Mistelbach, Austria; david.mutschlechner@mistelbach.lknoe.at (D.M.);; 2 Institute of Cardiovascular Pharmacotherapy and Interventional Cardiology, Karl Landsteiner Society, 3100 St. Pölten, Austria; 3 Karl Landsteiner University of Health Sciences, 3500 Krems, Austria; 4 Department of Internal Medicine, Cardiology and Nephrology, Universitätsklinikum Wiener Neustadt, 2700 Wiener Neustadt, Austria; 5 Institute of Vascular Medicine and Cardiac Electrophysiology, Karl Landsteiner Society, 3100 St. Pölten, Austria; 6 Department of Medicine, Faculty of Medicine and Dentistry, Danube Private University, 3500 Krems, Austria; 7 Department of Internal Medicine II, Medical University of Vienna, 1090 Vienna, Austria; 8 Department of Anesthesia and Intensive Care Medicine, Universitätsklinikum, 3100 St. Pölten, Austria; 9 Department of Blood Group Serology and Transfusion Medicine, Medical University of Vienna, 1090 Vienna, Austria

**Keywords:** platelet aggregation, sex, acute coronary syndrome, prasugrel, ticagrelor

## Abstract

**Background:** Dual antiplatelet therapy (DAPT) with a potent P2Y12 inhibitor is recommended for patients with acute coronary syndrome (ACS) following percutaneous coronary intervention (PCI). On-treatment platelet reactivity has been associated with ischemic endpoints and may vary between male and female patients. We, therefore, investigated sex-related differences in on-treatment platelet reactivity in ACS patients receiving ticagrelor or prasugrel. **Methods:** Maximal platelet aggregation by light-transmission aggregometry (LTA) and platelet surface P-selectin expression in response to arachidonic acid (AA), ADP, collagen, TRAP (a protease-activated receptor [PAR-1] agonist), and AYPGKF (a PAR-4 agonist) were assessed in 80 prasugrel- and 77 ticagrelor-treated patients 3 days after PCI. **Results:** In the overall study population (n = 157), women were older and had lower serum creatinine, hemoglobin, and hematocrit levels than men (all *p* < 0.05). Women exhibited higher ADP-inducible platelet aggregation in response to both 10 μM and 5 μM of ADP (both *p* < 0.05), while no sex-related differences were observed for AA-, TRAP-, collagen-, or AYPGKF-inducible platelet aggregation and agonist-inducible platelet surface P-selectin expression. In prasugrel-treated patients, women had higher ADP-inducible platelet aggregation and P-selectin expression compared with men (both *p* < 0.05), whereas no sex-related differences were found in ticagrelor-treated patients. In the multivariate linear regression analyses, female sex remained an independent predictor of higher platelet aggregation in response to 5 μM of ADP in prasugrel-treated patients (*p* < 0.05). High on-treatment residual platelet reactivity (HRPR) in response to AA was detected in four patients, and HRPR ADP was seen in seven patients, with no significant differences between female and male ACS patients (both *p* > 0.05). Low on-treatment residual platelet reactivity (LRPR) in response to AA was identified in 153 patients and LRPR ADP was present in 80 patients, with a higher prevalence of LRPR ADP in men (*p* = 0.01). **Conclusions:** Female ACS patients on prasugrel exhibited higher ADP-inducible platelet aggregation than male patients, while no sex-related differences were observed in patients on ticagrelor.

## 1. Introduction

Despite modern treatment options, acute coronary syndrome (ACS) remains the leading cause of death in the Western world [1]. Antithrombotic therapy is a key component of ACS management and its secondary prevention [2,3,4]. The P2Y12 receptor inhibitors ticagrelor and prasugrel have demonstrated superior effectiveness compared with clopidogrel in reducing the risk of future ischemic events in ACS patients [5,6,7]. Consequently, current ACS guidelines advise the use of prasugrel or ticagrelor as part of antithrombotic therapy after acute percutaneous coronary intervention (PCI) with stent implantation [8,9].

Ticagrelor and prasugrel achieve their antiplatelet effect by blocking the purinergic P2Y12 receptor found on the platelet surface. Under physiological conditions, this receptor is stimulated by adenosine diphosphate (ADP), which is released from dense granules during platelet activation, promoting further platelet aggregation and thrombus formation [2,4,10].

One primary mechanism behind ACS development is the rupture of an atherosclerotic plaque, which triggers local platelet activation and aggregation, leading to the formation of a thrombus and subsequent occlusion of a coronary artery [11]. Previous research has shown that female ACS patients who undergo PCI tend to have a worse prognosis than male ACS patients [12,13,14,15]. Several studies have demonstrated that women have a higher in-hospital and 30-day mortality compared with men [15,16]. Differences in baseline characteristics, as well as variations in plaque morphology, have been discussed as potential explanations for the seemingly worse short-term outcomes in female ACS patients [12].

However, sex-related differences in platelet activation and on-treatment platelet reactivity may also play a role in the higher rate of adverse events observed in female patients [11]. Prior studies have demonstrated an association between on-treatment platelet reactivity and ischemic endpoints across various cohorts of patients with cardiovascular diseases [17,18,19,20]. Moreover, previous investigations have documented that on-treatment platelet reactivity differs between female and male patients with coronary artery disease (CAD) [11,21,22]. In detail, female sex was linked to greater formation of leukocyte–platelet aggregates (LPAs) and enhanced PAR-1-mediated platelet reactivity in patients with atherosclerotic cardiovascular disease undergoing cardiac catheterization [11,21]. However, in ACS patients, sex has not been a determining factor in the choice of the P2Y12 inhibitor for dual antiplatelet therapy (DAPT) so far.

Data on sex-related differences in on-treatment platelet reactivity in ACS patients, particularly with regard to the specific P2Y12 inhibitor (ticagrelor or prasugrel), are very limited. We, therefore, compared on-treatment platelet reactivity between women and men in a cohort of ACS patients following PCI with stent implantation receiving DAPT with aspirin and either prasugrel or ticagrelor.

## 2. Materials and Methods

### 2.1. Study Population

The study cohort has been described previously [23]. In total, 157 ACS patients on daily aspirin (100 mg/day), and either prasugrel (10 mg/d or 5 mg/d in patients aged ≥ 75 years and those weighing < 60 kg; n = 80) or ticagrelor therapy (180 mg/d; n = 77) were included. Three days after successful PCI, blood was drawn after an overnight fast. Before collecting the blood samples, the attending physician had to ensure that the patient had correctly taken the last dose of DAPT the previous day and that the samples were drawn before the morning medication. The time interval between the last administration of prasugrel or ticagrelor and blood sampling was similar in all patients. As previously mentioned, the exclusion criteria comprised oral anticoagulation with either vitamin K antagonists (warfarin, phenprocoumon, or acenocoumarol) or direct oral anticoagulants (edoxaban, dabigatran, apixaban, or rivaroxaban), a known aspirin, prasugrel or ticagrelor intolerance (allergic reactions or gastrointestinal bleeding complications), a history of bleeding disorders, treatment with ticlopidine, dipyridamol or nonsteroidal anti-inflammatory drugs, malignant myeloproliferative disorders or heparin-induced thrombocytopenia, major surgery within one week before enrollment, severe hepatic failure, known qualitative defects in platelet function, a platelet count of <100,000 or >450,000/µL, and a hematocrit level of <30% [23].

The study protocol was in accordance with the Declaration of Helsinki and was approved by the Ethics Committee of the Medical University of Vienna (protocol code: 1940/2013; date of approval: 11 November 2013). All participants gave their written informed consent.

### 2.2. Blood Sampling

Blood was drawn by aseptic venipuncture from an antecubital vein using a 21-gauge butterfly needle (0.8 × 19 mm; Greiner Bio-One, Kremsmünster, Austria) as previously described [23,24]. To avoid procedural deviations, all blood samples were taken by the same physician applying a light tourniquet that was immediately released, and the samples were mixed by gently inverting the tubes. Whole blood was drawn into citrate-anticoagulated tubes (Greiner Bio-One, Kremsmünster, Austria) after an overnight fast. All specimens were manually brought to the laboratory by the collecting physician immediately after blood sampling. Platelet function testing was performed within 30 min in all patients without further time delay (median: 25 min; IQR: 22–28 min). This procedure was uniformly conducted for all enrolled patients by the same physician, ensuring a high level of procedural consistency.

### 2.3. Light-Transmission Aggregometry (LTA)

LTA was performed with a PAP-8E aggregometer (Bio-Data, Horsham, PA, USA) as previously described [25,26]. Citrate-anticoagulated whole blood was allowed to “rest” in a tilt position at room temperature for 20 min before centrifugation. Blood tubes were centrifuged at 150× *g* for 10 min to acquire platelet-rich plasma (PRP).

To obtain platelet-poor plasma (PPP), the remaining specimens were re-centrifugated at 2000× *g* for 10 min. Platelet counts were not adjusted as the median platelet count was 238 G/L (range 150–375 G/L). Platelet aggregation was initiated by the addition of arachidonic acid (AA; 1600 μM; Roche Diagnostics, Mannheim, Germany), ADP (5 μM; Roche Diagnostics), ADP (10 μM; Roche Diagnostics), collagen (2.7 μg/mL; Roche Diagnostics), thrombin receptor-activating peptide (TRAP; a PAR-1 agonist, 25 μM; Bachem, Bubendorf, Switzerland), or AYPGKF (a PAR-4 agonist, 645 μΜ; Roche Diagnostics) as agonists to PRP. Optical density changes were recorded photoelectrically for 10 min as platelets began to aggregate to obtain the maximal aggregation %. The maximal aggregation % was automatically calculated by the PAP-8E aggregometer by comparing the increase in light transmission through platelet-rich plasma after the addition of an agonist to the baseline optical density that was set with PPP and considered as 100% platelet aggregation.

### 2.4. Determination of Platelet Surface P-Selectin

The expression of P-selectin was determined in citrate-anticoagulated blood, as previously described [27]. In brief, whole blood was diluted in phosphate-buffered saline to obtain 20 × 10^3^/μL of platelets in 20 μL and incubated for 10 min with the platelet-specific monoclonal antibody anti-CD42b (clone HIP1, allophycocyanin-labeled; Becton Dickinson (BD), San Jose, CA, USA), without agonists, and after in vitro exposure to suboptimal concentrations of AA (final concentration: 80 μM; Roche Diagnostics), ADP (final concentration: 1 μM; Roche Diagnostics), TRAP (final concentration: 14.25 μM; Bachem), or AYPGKF (final concentration: 714 μM; Roche Diagnostics), each 10 μL, for 10 min. The concentrations of these agonists were established in preliminary titration experiments involving 10 healthy individuals, where dose–response curves were generated. The selected concentrations corresponded to approximately 60–70% of the maximum achievable increase in median fluorescence intensity (MFI) in healthy controls.

Following agonist stimulation, the samples were further incubated for 10 min with an antibody mix targeting P-selectin (anti-CD62p-phycoerythrin, clone CLB-Thromb6; Immunotech, Beckman Coulter, Marseille, France). Isotype-matched control antibodies (BD) were utilized to assess nonspecific binding. To stop the reaction, 500 μL of PBS was added, and the samples were immediately analyzed using a FACSCanto II flow cytometer (BD). Platelets were identified based on their characteristic forward-scatter versus side-scatter profiles, with 10,000 events recorded within this predefined gate. Further discrimination of platelets was achieved by assessing anti-CD42b staining in conjunction with side-scatter characteristics. P-selectin binding was quantified using histogram analysis. To ensure measurement accuracy, daily calibration of the flow cytometer was conducted using cytometer setup and tracking beads (BD), with data acquisition managed via the Diva software. The MFI based on all events, as well as the percentage of P-selectin-positive cells, was used for statistical calculations [27].

### 2.5. Statistical Analysis

All continuous variables are expressed as medians (interquartile range (IQR): 25th–75th percentiles) or means (±standard deviations). Categorical variables are reported as numbers (percentages). Comparisons of continuous variables between two groups were performed using the Mann–Whitney U test, while comparisons involving more than two groups were performed using the Kruskal–Wallis test. Chi-square (χ^2^) tests were used to compare categorical variables. For the multivariate linear regression model, preliminary univariate regression analyses were performed for all baseline characteristics. Covariates that were statistically significant in the univariate analysis were considered as potential confounders and included in the multivariate regression model. The robustness of the multivariate models was assessed through the evaluation of residual plots and variance inflation factors to exclude collinearity. As the primary outcome variables were non-normally distributed, a sensitivity analysis using log-transformed aggregation values was also performed but did not materially alter the results. Model fit was assessed using the coefficient of determination (adjusted R^2^). Regression coefficients (B), 95% confidence intervals (CIs), and *p*-values were reported for each model. The detailed results of the univariate and multivariate regression analyses are provided in the Supplementary Tables. Boxplots were generated to visually represent continuous variables, with *p*-values displayed for sex-based comparisons within each P2Y12 inhibitor group. A two-tailed *p* ≤ 0.05 was considered statistically significant. All statistical analyses were performed using SPSS 29.0.2 (Armonk, NY, USA) and Python 3.13.5 (Seaborn, SciPy, Matplotlib, and Pandas libraries; Dover, DE, USA). 

## 3. Results

### 3.1. Baseline Characteristics

A total of 157 ACS patients on DAPT with aspirin and either prasugrel (n = 80) or ticagrelor (n = 77) were included in this study. The baseline characteristics of the study population stratified by sex are presented in Table 1. The women were significantly older than the men (62.8 ± 12.2 years vs. 57.5 ± 10.8 years; *p* = 0.02), had lower serum creatinine levels (0.8 mg/dL [0.7–0.9 mg/dL] vs. 1.0 mg/dL [0.9–1.1 mg/dL]; *p* < 0.001), lower hemoglobin levels (12.5 g/dL [11.6–13.1 g/dL] vs. 14.1 g/dL [13.3–14.9 g/dL]; *p* < 0.001), and lower hematocrit levels (36.6% [34.2–38.3%] vs. 41.6% [39.1–44.0%]; *p* < 0.001). No significant sex-related differences were observed in body mass index (BMI), cardiovascular risk factors, or medication.

### 3.2. Platelet Aggregation and Platelet Surface P-Selectin Expression According to Sex

The LTA results and platelet surface P-selectin expression, stratified by sex, are presented in Table 2. Agonist-inducible platelet surface P-selectin expression between women and men was comparable in the overall patient population (all *p* > 0.05; Table 2). In contrast, women exhibited significantly higher ADP-inducible platelet aggregation than men (38.5% [31.0–45.8%] vs. 34.0% [25.0–41.0%]; *p* = 0.03) in response to 10 μM of ADP, as well as higher ADP-inducible platelet aggregation (30.5% [24.0–37.75%] vs. 24.0% [19.0–31.0%]; *p* < 0.001) in response to 5 μM of ADP. However, no significant sex-related differences in AA-, collagen-, TRAP-, or AYPGKF-inducible platelet aggregation were detectable in the overall patient population (all *p* > 0.05; Table 2).

High residual platelet reactivity (HRPR) in response to AA and ADP was determined based on prior research linking platelet aggregation measured by LTA to ischemic events after PCI [28,29]. The defined cutoff values for HRPR were a maximal aggregation of ≥20% for LTA AA and ≥70% for LTA ADP (10 µM). Applying these thresholds, HRPR AA was identified in 4 patients (2.5%), while HRPR ADP was observed in 7 patients (4.5%). The HRPR prevalence was comparable between female and male patients (both *p* > 0.05; Table 2). Moreover, the prevalence of HRPR was comparable between patients on prasugrel vs. ticagrelor (HRPR AA: 2.5% vs. 2.6%, *p* = 1.0; HRPR ADP: 5.0% vs. 3.9%, *p* = 0.7).

Low on-treatment residual platelet reactivity (LRPR) in response to AA and ADP was determined based on prior research linking LRPR by LTA to bleeding events after PCI [30,31]. The defined cutoff values for LRPR were a maximal aggregation of ≤20% for LTA AA and <25.5% for LTA ADP (5 µM). Applying these thresholds, LRPR AA was identified in 153 patients (97.5%), while LRPR ADP was observed in 80 patients (51.0%). Thereby, the prevalence of LRPR AA was comparable between female and male patients (*p* = 0.8), while LRPR ADP was seen more frequently in men compared with women (56.0% vs. 31.3%, *p* = 0.01; Table 2). In addition, LRPR AA was observed in 78 patients (97.5%) on prasugrel and 75 patients (97.4%) on ticagrelor, while LRPR ADP was observed in 44 patients (55.0%) on prasugrel and 36 patients (46.8%) on ticagrelor, respectively. The prevalence of LRPR was comparable between patients on prasugrel and patients on ticagrelor (LRPR AA: 97.5% vs. 97.4%, *p* = 1.0; LRPR ADP: 55.0% vs. 46.8%, *p* = 0.3).

### 3.3. Platelet Aggregation and Platelet Surface P-Selectin Expression According to P2Y12 Inhibitor

**Figure 1:**
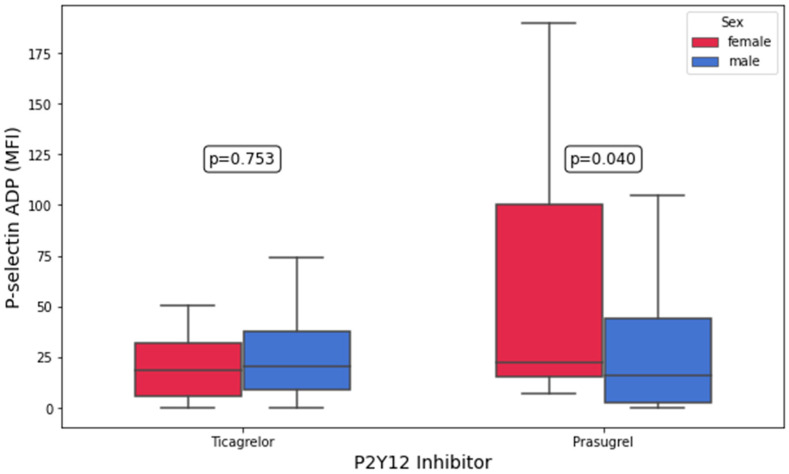
Platelet surface P-selectin expression in response to adenosine diphosphate (ADP) in patients on ticagrelor and prasugrel stratified by sex.

On-treatment platelet aggregation and platelet surface P-selectin expression in patients receiving ticagrelor vs. prasugrel, stratified by sex, are presented in Table 3. Platelet surface P-selectin expression after ADP stimulation (22.3 MFI [15.1–100.1 MFI] vs. 15.8 MFI [2.6–44.0 MFI], *p* = 0.04; Figure 1) and AA stimulation (7.8% [5.5–13.8%] vs. 5.4% [3.5–9.9%], *p* = 0.05) was significantly higher in prasugrel-treated women compared with prasugrel-treated men, whereas no sex-related differences were observed in ticagrelor-treated patients. However, in both the prasugrel- and ticagrelor-treated subgroups, no differences in TRAP- and AYPGKF-inducible platelet surface P-selectin expression between female and male patients were observed (all *p* > 0.05). Nonetheless, in the univariate linear regression, no significant association was found between sex and P-selectin expression following ADP (B = 27.380; *p* = 0.053) and AA stimulation (B = 5.843 and *p* = 0.2; both in Table 4 and Supplementary Table S1). The adjusted R^2^ values for the models predicting P-selectin expression following ADP and AA stimulation were 0.050 and 0.024, respectively, indicating a limited proportion of explained variance.

**Figure 2:**
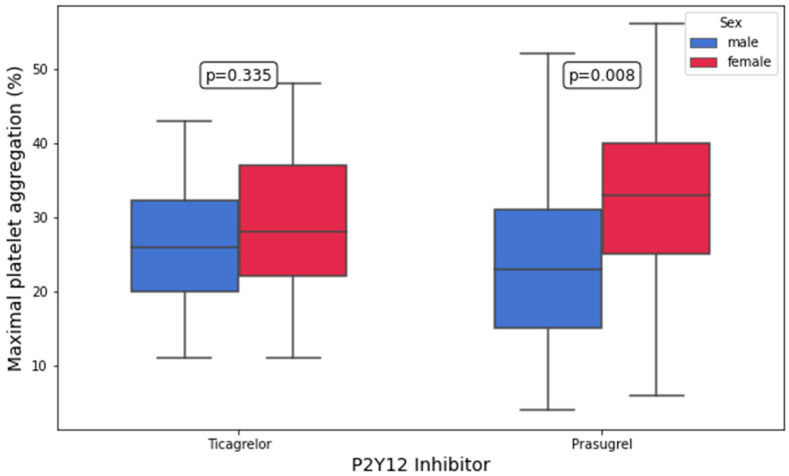
Maximal platelet aggregation (%) in response to 5 µM of adenosine diphosphate (ADP) by light-transmission aggregometry (LTA) in patients on ticagrelor and prasugrel stratified by sex.

**Figure 3:**
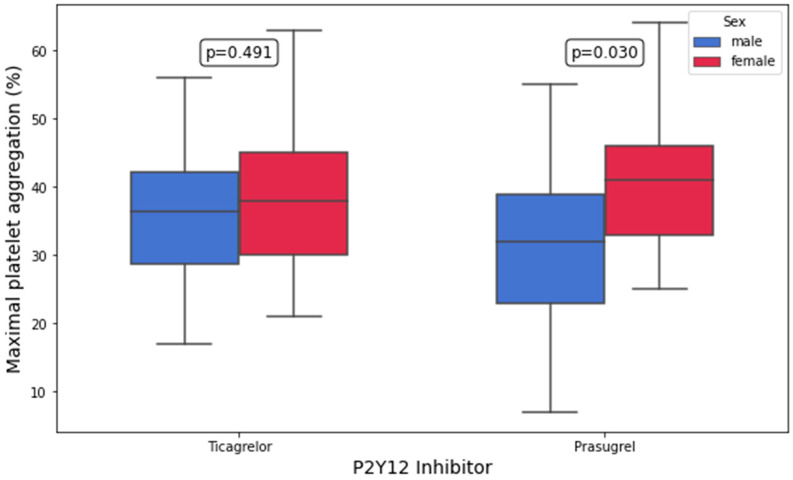
Maximal platelet aggregation (%) in response to 10 µM of adenosine diphosphate (ADP) by light-transmission aggregometry (LTA) in patients on ticagrelor and prasugrel stratified by sex.

Platelet aggregation by LTA in response to 10 μM of ADP and 5 μM of ADP was significantly higher in prasugrel-treated women compared with prasugrel-treated men (10 μM of ADP: 41.0% [33.0–46.0%] vs. 32.0% [23.0–39.0%], *p* = 0.03; 5 μM of ADP: 33% [25–40%] vs. 23.0% [15–31%], *p* = 0.008; Figure 2 and Figure 3). In contrast, no significant differences in AA-, collagen-, TRAP, and AYPGKF-inducible platelet aggregation between women and men were found in either treatment group (all *p* > 0.05; Table 3). In the multivariate linear regression analyses, female sex remained an independent predictor of higher platelet aggregation following stimulation with 5 μM of ADP in prasugrel-treated ACS patients (B = 20.368, 95% CI: 5.325 to 35.410, *p* = 0.009; Table 4 and Supplementary Table S2). Notably, female sex was associated with higher platelet aggregation upon stimulation with 10 μM of ADP in the univariate analysis, but this association did not remain significant in the multivariate modeling (B = 10.712, 95% CI: −2.536 to 23.960, *p* = 0.1; Table 4 and Supplementary Table S3). The models predicting ADP-inducible platelet aggregation showed adjusted R^2^ values of 0.178 (10 μM) and 0.307 (5 μM), suggesting a moderate-to-substantial explanatory capacity of the selected predictors, particularly for low-dose ADP stimulation.

## 4. Discussion

In this study, we investigated sex-related differences in on-treatment platelet reactivity and platelet surface P-selectin expression in ACS patients on DAPT with either prasugrel or ticagrelor. Overall, female ACS patients exhibited higher ADP-inducible platelet aggregation, as assessed by LTA, compared with male patients. Among patients on prasugrel, ADP-inducible platelet aggregation was significantly higher in women than in men. Additionally, platelet surface P-selectin expression following ADP and AA stimulation was significantly higher in prasugrel-treated female patients in univariate analyses. In contrast, in patients on ticagrelor, platelet aggregation and platelet surface P-selectin expression in response to all analyzed agonists did not differ significantly between female and male patients.

In this study, we used LTA as the ‘historical gold standard’ for measuring platelet aggregation [32,33]. This approach was chosen because LTA has repeatedly been associated with clinical outcomes following PCI [34]. Additionally, our team previously found significantly higher levels of PAR-1-mediated platelet aggregation by LTA in female compared with male patients undergoing elective angioplasty with stent implantation [11,21]. However, the limitations of LTA are well-documented and should not be overlooked. As previously mentioned, LTA requires multiple centrifugation steps, which introduces a degree of operator dependence compared with other platelet function tests, such as multiple electrode aggregometry (MEA). MEA is a well-standardized and, therefore, reproducible platelet function test. It uses the principle of impedance aggregometry to assess platelet function in diluted whole blood, with the advantage of preserving the natural cellular environment in order to approximate in vivo platelet aggregation. However, physiological factors such as blood flow dynamics or endothelial influences cannot be captured by MEA [32,33].

Moreover, we measured platelet surface P-selectin as a sensitive marker of platelet activation. During platelet activation, P-selectin is released from alpha granules and translocated to the platelet surface, where it plays a key role in intercellular interactions. It binds to GPIbα, promoting platelet–platelet adhesion, and serves as the primary ligand for the P-selectin glycoprotein ligand-1 receptor on leukocytes, facilitating leukocyte–platelet interactions [35,36]. However, using flow cytometric platelet function analysis, measuring platelet surface P-selectin expression is not without limitations. It requires immediate processing of samples to avoid pre-analytical platelet activation, which can confound data interpretation. Furthermore, variability in antibody clones, fluorochrome conjugation, titration, and gating strategies leads to poor inter-laboratory comparability unless harmonized protocols are used, which is currently lacking in many clinical settings [37,38,39].

Previous studies have consistently reported sex-related differences in platelet function, with women exhibiting higher platelet reactivity and a greater predisposition to thrombosis compared with men [40,41]. These differences have been attributed to biological factors, including hormonal influences, variations in platelet receptor expression, and differences in antithrombotic drug metabolism. Additionally, previous research has demonstrated that women exhibit enhanced leukocyte–platelet interactions, which may further contribute to increased platelet reactivity and thrombotic risk [11]. In detail, we found that female patients with atherosclerosis displayed significantly higher LPA than male patients, potentially pointing toward a prothrombotic milieu despite conventional antiplatelet therapy [11]. In another study in 562 patients undergoing cardiac catherization, we observed more pronounced PAR-1 signaling in women compared with men without ACS [21]. In line with the current study, these differences were not seen in ACS patients. Together with the present data, these previous findings suggest differences in PAR-1-mediated platelet responses between women with stable CAD and those presenting with ACS.

To the best of our knowledge, this is the first study to demonstrate higher on-treatment platelet aggregation in response to ADP in women with ACS receiving DAPT with a potent P2Y12 inhibitor. Notably, the observed difference between prasugrel and ticagrelor is of particular interest. While ticagrelor-treated women and men showed comparable on-treatment platelet aggregation by LTA and similar platelet surface P-selectin expression, prasugrel-treated women exhibited higher ADP-inducible platelet aggregation and platelet surface P-selectin expression, indicating that prasugrel may be less effective in mitigating platelet activation in women compared with men with ACS.

These findings are consistent with the results of a subgroup analysis of the ISAR-REACT 5 trial [42]. In this trial, prasugrel demonstrated superior efficacy over ticagrelor in reducing ischemic events. However, this benefit was primarily observed in men, while no significant difference between the two agents was found in women [42]. Our findings provide a potential mechanistic explanation for this observation: higher residual platelet reactivity and increased P-selectin expression in response to ADP in prasugrel-treated women may offset the expected ischemic benefit compared with male patients. Given that P-selectin plays a key role in leukocyte–platelet interactions and thrombus formation, its increased expression in prasugrel-treated women may reflect a less pronounced antithrombotic effect of prasugrel in female ACS patients. However, it must be noted that in the multivariate linear regression analysis, no significant association was found between sex and ADP-inducible P-selectin expression as well as platelet aggregation following stimulation with 10 μM of ADP in prasugrel-treated patients. Moreover, we did not observe a difference in the prevalence of HRPR between female and male ACS patients in our study, while LRPR ADP was significantly higher in male than in female patients.

The clinical implications of our findings warrant further investigation. On-treatment platelet reactivity has been associated with an increased risk of thrombotic events despite guideline-recommended antiplatelet therapy [43]. While our findings suggest potential sex differences in the antiplatelet effects of prasugrel in ACS, further research is necessary to determine whether these differences translate into clinical outcomes. If a reduced clinical efficacy of prasugrel in women was confirmed in large outcome trials, individualized treatment strategies, such as alternative antiplatelet regimens or dose adjustments, should be evaluated in order to optimize therapy. Moreover, our results emphasize the importance of considering sex-specific differences in platelet function when evaluating and prescribing antiplatelet therapies.

Our key findings indicate that while ticagrelor-treated ACS patients exhibit no significant sex-related differences in on-treatment platelet aggregation, female ACS patients on prasugrel show significantly higher ADP-inducible platelet aggregation compared with prasugrel-treated male ACS patients. These results may suggest that prasugrel is less effective in inhibiting platelet activation in women, potentially impacting its clinical benefit in this subgroup. Further studies with larger cohorts and clinical outcome assessments are needed to validate our findings and explore tailored antiplatelet strategies for female ACS patients.

### Limitations

Our study has several limitations. First, all data were derived from a single-center cohort. Therefore, our findings require validation in independent, larger, multicenter studies. Second, the choice of the P2Y12 antagonist was made by the treating physician, which may have led to patient selection bias. However, the two treatment groups were well-matched, and there were no significant differences regarding the most relevant patient characteristics. Third, while we observed significant differences in on-treatment platelet aggregation between women and men, clinical outcome data were not available to determine whether these differences translated into higher rates of ischemic events. Fourth, due to missing variables (history of bleeding events), we cannot provide the exact HAS-BLED score for all patients of our study cohort in order to assess the risk of bleeding events. However, as shown in Table 1, the prevalence of a HAS-BLED score of ≥3 was comparable between female and male patients. Fifth, when interpreting our findings, it should be noted that no pharmacogenetic analyses were performed in the investigated cohort. Sixth, our sample size was limited, which may affect the generalizability of our findings. Finally, while measurement of platelet surface P-selectin expression provides a reliable marker of platelet activation, additional markers such as leukocyte–platelet aggregates could further elucidate sex-specific differences in thrombotic risk.

## 5. Conclusions

In conclusion, our study highlighted sex-related differences in on-treatment platelet aggregation and activation among prasugrel-treated ACS patients, with women demonstrating higher ADP-inducible platelet aggregation despite DAPT with a potent P2Y12 inhibitor. These findings may have important implications for the optimization of antiplatelet strategies in women with ACS and call for further studies to assess whether tailored approaches can improve outcomes in female ACS patients.

## Figures and Tables

**Table 1:** Baseline characteristics of the study cohort. Data are presented as means ± standard deviations (SDs) for normally distributed continuous variables, medians (Q1–Q3) for non-normally distributed continuous variables, and counts (percentages (%)) for categorical variables. Abbreviations: ACE, angiotensin-converting enzyme; ARB, angiotensin II receptor blocker; CRP, C-reactive protein; TIA, transient ischemic attack.

Characteristic	Women (n = 32)	Men (n = 125)	*p*
Demographics			
Age, years	62.8 ± 12.2	57.5 ± 10.8	0.02
Caucasian, white No. (%)	32 (100)	125 (100)	1.0
Body mass index, kg/m^2^	29.2 ± 5.4	28.4 ± 4.6	0.5
Medical history			
Prior myocardial infarction, No. (%)	5 (16)	22 (18)	0.9
Prior stroke or TIA, No. (%)	0 (0)	5 (4)	0.5
Arterial hypertension, No. (%)	21 (66)	86 (71)	0.7
Hyperlipoproteinemia, No. (%)	25 (81)	91 (76)	0.7
Peripheral artery disease, No. (%)	2 (6)	12 (10)	0.7
Diabetes mellitus type II, No. (%)	10 (32)	30 (25)	0.5
Smoking, No. (%)	17 (52)	92 (74)	0.3
HAS-BLED score ≥ 3, No. (%)	21 (65.6)	86 (68.8)	0.7
Laboratory data			
Serum creatinine, mg/dL	0.8 (0.7–0.9)	1.0 (0.9–1.1)	<0.001
Platelet count, G/L	229 (203–279)	226 (187–251)	0.2
Hemoglobin, g/dL	12.5 (11.6–13.1)	14.1 (13.3–14.9)	<0.001
Hematocrit, %	36.6 (34.2–38.3)	41.6 (39.1–44.0)	<0.001
Highly sensitive CRP, mg/dL	1.4 (0.8–3.9)	1.2 (0.6–3.4)	0.7
Medication			
Aspirin, No. (%)	32 (100)	125 (100)	1.0
Prasugrel, No (%)	15 (46.9)	65 (52)	0.6
Ticagrelor, No. (%)	17 (53.1)	60 (48)	0.8
Statin, No. (%)	32 (100)	121 (98)	1.0
Beta-blocker, No. (%)	32 (100)	118 (95.9)	0.6
ACE inhibitor or ARB, No. (%)	28 (90.3)	121 (98.4)	0.1
Calcium-channel blocker, No. (%)	6 (19)	9 (7)	0.1
SGLT2 inhibitor, No. (%)	2 (6.3)	3 (2.4)	0.3

**Table 2:** Platelet surface P-selectin expression and platelet aggregation measured by light-transmission aggregometry (LTA), stratified by sex. Platelet aggregation by LTA is reported as maximal aggregation %, while platelet surface P-selectin expression is reported as percentage (%) of P-selectin-positive cells and median fluorescence intensity (MFI). Data are expressed as medians (Q1–Q3). Abbreviations: AA, arachidonic acid; ADP, adenosine diphosphate; COL, collagen; HRPR, high on-treatment residual platelet reactivity; LRPR, low on-treatment residual platelet reactivity; LTA, light-transmission aggregometry; TRAP, thrombin receptor-activating peptide.

Parameter	Women (n = 32)	Men (n = 125)	*p*
HRPR AA (LTA ≥ 20% to AA)	2 (6)	2 (1.6)	1
HRPR ADP (LTA ≥ 70% to 10 μM of ADP)	2 (6)	5 (4)	0.9
LRPR AA (LTA ≤ 20% to AA)	31 (96.9)	122 (97.6)	0.8
LRPR ADP (LTA < 25.5% to 5 μM of ADP)	10 (31.3)	70 (56.0)	0.01
P-selectin, ADP (MFI)	22.0 (11.5–50.5)	17.7 (5.0–38.8)	0.3
P-selectin, ADP (%)	11.3 (9.6–18.8)	11.7 (7.6–17.0)	0.4
P-selectin, AA (MFI)	0.6 (0.0–17.1)	0.2 (0.0–13.0)	0.8
P-selectin, AA (%)	5.1 (4.0–8.2)	5.4 (3.4–9.1)	0.6
P-selectin, TRAP (MFI)	2630.7 (1775.2–3228.2)	2993.2 (1907.4–3915.2)	0.3
P-selectin, TRAP (%)	84.7 (79.5–88.9)	87.5 (80.2–92.5)	0.2
P-selectin, AYPGKF (MFI)	188.3 (90.7–554.9)	208.6 (92.7–741.7)	0.3
P-selectin, AYPGKF (%)	39.7 (23.7–60.2)	43.1 (31.4–64.8)	0.2
ADP-inducible platelet aggregation, 10 μM of ADP (%)	38.5 (31.0–45.75)	34.0 (25.0 −41.0)	0.03
ADP-inducible platelet aggregation, 5 μM of ADP (%)	30.5 (24.0–37.75)	24.0 (19.0–31.0)	<0.001
AA-inducible platelet aggregation (%)	3 (2–6.25)	2.0 (1.0–4.0)	0.3
COL-inducible platelet aggregation (%)	75.5 (37.5–93.75)	70 (44–82)	0.5
TRAP-inducible platelet aggregation (%)	80 (63.75–95.5)	72.0 (61.0–86.0)	0.1
AYPGKF-inducible platelet aggregation (%)	70 (58.0–90.0)	71.5 (56.0–86.0)	0.5

**Table 3:** Comparison of platelet surface P-selectin expression and maximal platelet aggregation between ticagrelor- and prasugrel-treated patients, further stratified by sex. Platelet aggregation by LTA is reported as maximal aggregation %, while platelet surface P-selectin expression is reported as percentage (%) of P-selectin-positive cells and median fluorescence intensity (MFI). Data are presented as medians (Q1–Q3). Abbreviations: AA, arachidonic acid; ADP, adenosine diphosphate; COL, collagen; LTA, light-transmission aggregometry; TRAP, thrombin receptor-activating peptide.

	Ticagrelor (n = 77)			Prasugrel (n = 80)		
Parameter	Women (n = 16)	Men (n = 61)	*p*	Women (n = 16)	Men (n = 64)	*p*
P-selectin, ADP (MFI)	18.4 (5.8–31.9)	20.6 (8.7–37.4)	0.8	22.3 (15.1–100.1)	15.8 (2.6–44.0)	0.04
P-selectin, ADP (%)	10.2 (8.5–15.6)	11.8 (8.7–16.5)	0.6	11.8 (10.1–30.7)	11.7 (7.1–18.0)	0.09
P-selectin, AA (MFI)	0.0 (0.0–3.0)	1.4 (0.0–11.7)	0.3	10.4 (0.0–20.4)	0.0 (0.0–13.3)	0.1
P-selectin, AA (%)	4.7 (3.2–5.0)	5.4 (3.1–8.1)	0.3	7.8 (5.5–13.8)	5.4 (3.5–9.9)	0.05
P-selectin, TRAP (MFI)	2380.2 (1835.7–2718.2)	2719.2 (1629.8–3625.5)	0.4	3151.3 (1829.6–3810.4)	3367.5 (2272.0–4107.3)	0.7
P-selectin, TRAP (%)	84.5 (79.8–86.9)	87.0 (78.9–90.6)	0.3	86.8 (73.3–94.1)	90.1 (82.5–93.8)	0.6
P-selectin, AYPGKF (MFI)	168.1 (74.3–426.7)	187.4 (88.8–607.2)	0.7	248.5 (90.7–636.8)	291.7 (104.9–889.2)	0.3
P-selectin, AYPGKF (%)	40.2 (23.8–52.9)	41.7 (29.6–62.0)	0.6	37.1 (23.1–61.1)	47.5 (32.6–68.2)	0.3
ADP-inducible platelet aggregation, 10 μM of ADP (%)	38.0 (30.0–45.0)	36.5 (28.8–42.2)	0.5	41.0 (33.0–46.0)	32.0 (23.0–39.0)	0.03
ADP-inducible platelet aggregation, 5 μM of ADP (%)	28.0 (22.0–37.0)	26.0 (20.0–32.2)	0.3	33.0 (25.0–40.0)	23.0 (15.0–31.0)	0.008
AA-inducible platelet aggregation (%)	2.0 (2.0–5.0)	3.0 (2.0–5.2)	0.8	4.0 (2.0–6.5)	2.0 (1.0–4.0)	0.1
COL-inducible platelet aggregation (%)	66.0 (38.0–83.0)	74.0 (56.0–83.0)	0.5	79.0 (35.0–97.0)	55.0 (27.0–81.0)	0.2
TRAP-inducible platelet aggregation (%)	80.0 (64.0–94.0)	73.0 (60.8–85.0)	0.2	79.0 (63.0–95.0)	72.0 (61.0–87.0)	0.4
AYPGKF-inducible platelet aggregation (%)	65.0 (58.5–78.0)	72.5 (61.8–86.0)	0.6	85.0 (58.0–96.0)	71.5 (55.0–86.5)	0.1

**Table 4:** Multivariate linear regression analysis for patients on prasugrel. All models adjusted for univariate significant covariates. Abbreviations: AA, arachidonic acid; ADP, adenosine diphosphate; B, regression coefficient; MFI, median fluorescence intensity.

Parameter	Variable	B (Unstandardized Coefficient)	95% Confidence Interval	*p*
P-selectin, ADP (MFI)	Female vs. male	27.380	−0.378–55.138	0.053
P-selectin, AA (MFI)	Female vs. male	5.843	−2.736–14.423	0.179
ADP-inducible platelet aggregation, 5 μM of ADP (%)	Female vs. male	20.368	5.325–35.410	0.009
ADP-inducible platelet aggregation, 5 μM of ADP (%)	Age	0.339	−0.105–0.783	0.131
ADP-inducible platelet aggregation, 5 μM of ADP (%)	Hemoglobin	0.780	−11.359–12.919	0.898
ADP-inducible platelet aggregation, 5 μM of ADP (%)	Hematocrit	−0.265	−4.526–3.995	0.901
ADP-inducible platelet aggregation, 5 μM of ADP (%)	Creatinine	−5.183	−39.948–20.582	0.687
ADP-inducible platelet aggregation, 5 μM of ADP (%)	Highly sensitive CRP	−0.300	−1.742–1.141	0.677
ADP-inducible platelet aggregation, 5 μM of ADP (%)	SGLT2 inhibitor	−1.041	−32.413–30.331	0.947
ADP-inducible platelet aggregation, 10 μM of ADP (%)	Female vs. male	10.712	−2.536–23.960	0.111
ADP-inducible platelet aggregation, 10 μM of ADP (%)	Age	0.166	−0.234–0.567	0.410
ADP-inducible platelet aggregation, 10 μM of ADP (%)	Hemoglobin	5.984	−5.545–17.514	0.303
ADP-inducible platelet aggregation, 10 μM of ADP (%)	Hematocrit	−2.439	−6.410–1.531	0.224
ADP-inducible platelet aggregation, 10 μM of ADP (%)	SGLT2 inhibitor	9.122	−23.670–41.913	0.580

## Data Availability

The data underlying this study contain sensitive clinical information and are, therefore, not publicly available in order to protect the privacy and confidentiality of the study participants. Access to these data is restricted in accordance with institutional policies and applicable data protection regulations. Qualified researchers may request access to the anonymized data, subject to review and approval by the corresponding ethics committee and the data-holding institution.

## Supplementary Materials

The following supporting information can be downloaded at https://www.mdpi.com/article/10.3390/biomedicines13092068/s1, Table S1: Univariate linear regression analyses including all baseline characteristics as covariates for platelet surface P-selectin expression after arachidonic acid (left column) and ADP stimulation (right column) in patients on prasugrel. Table S2: Uni- and multivariate linear regression analyses including all baseline characteristics as covariates for platelet aggregation in response to 5 μM of ADP measured by light-transmission aggregometry (LTA) in patients on prasugrel. Table S3: Uni- and multivariate linear regression analyses including all baseline characteristics as covariates for platelet aggregation in response to 10 μM of ADP measured by light-transmission aggregometry (LTA) in patients on prasugrel.
