# Supplementary material for: Sex-Related Differences in On-Treatment Platelet Reactivity in Patients with Acute Coronary Syndrome
Source: Biomedicines. 2025 Aug 25;13(9):2068. doi: 10.3390/biomedicines13092068 (PMC12467199; doi:10.3390/biomedicines13092068)
Supplement: Supplementary file 1 [file biomedicines-13-02068-s001.zip › biomedicines-3829056-supplementary.pdf]

Supplementary Table S1: Univariate linear regression analyses including all baseline characteristics as covariates for platelet surface P-selectin expression after arachidonic acid (left column) and ADP stimulation (right column) in patients on prasugrel. Abbreviations: ADP, adenosine diphosphate; ACE, angiotensin-converting enzyme; ARB, angiotensin receptor blocker; BMI, body mass index;., B, regression coefficient; CI, confidence interval; CRP, C-reactive protein; TIA, transient ischemic attack.

**Covariates**	**P-Selectin expression after AA stimulation**			**P-Selectin expression after ADP stimulation**		
	**B (CI 95 %)**	**Beta**	***p***	**B (CI 95 %)**	**Beta**	***p***
Age	0.145 (-0.120 – 0.411)	0.103	0.281	0.631 (-0.106 – 1.368)	0.160	0.093
Female Sex	5.843 (-2.736 – 14.423)	0.156	0.179	27.380 (-0.378 – 55.138)	0.223	0.053
BMI	0.188 (-0.332 – 0.707)	0.059	0.476	1.259 (-0.175 – 2.693)	0.143	0.085
Prior mycardial infarction	-2.018(-8.164 – 4.128)	-0.054	0.517	-11.134 (-28.157 – 5.888)	-0.107	0.198
Piror stroke or TIA	-3.121 (-16.871 – 10.629)	-0.037	0.654	-1.940 (-40.027 – 36.147)	-0.008	0.920
Arterial hypertension	-1.932 (-7.255 – 3.391)	-0.059	0.474	3.599 (-11.150 – 18.348)	0.040	0.630
Hyperlipoproteinemia	1.473 (-4.446 – 7.392)	0.041	0.624	-4.512 (-20.970 – 11.947)	-0.045	0.589
Peripheral artery disease	- 0.002 (-7.992 – 7.989)	0.0	1.0	-2.945 (-25.185 – 19.296)	-0.022	0.794
Diabetes mellitus type II	-2.526 (-9.867 – 4.815)	-0.077	0.495	-4.592 (-28.310 – 19.126)	-0.043	0.701
Smoking	-0.734 (-3.913 – 2.445)	-0.038	0.649	-3.207 (-12.042 – 5.628)	-0.060	0.474
HAS-BLED Score ≥3	-1.932 (-7.255 – 3.391)	-0.059	0.474	3.599 (-11.150 – 18.348)	0.040	0.630
Serum creatinine	-1.722 (-9.711 – 6.266)	-0.037	0.670	-1.434 (-21.582 – 18.714)	-0.012	0.888
Platelet count, G/l	-0.027 (-0.069 – 0.015)	-0.103	0.214	-0.058 (-0.175 – 0.059)	-0.081	0.327
Hemoglobin	-0.251 (-1.892 – 1.390)	-0.025	0.763	-3.765 (-8.292 – 0.763)	-0.135	0.102
Hematocrit	-0.081 (-0.651 – 0.488)	-0.023	0.778	-1.620 (-3.183 - 0.056)	-0.168	0.052
High sensitivity CRP	-0.206 (-0.824 – 0.411)	-0.056	0.510	0.192 (-1.532 – 1.916)	0.019	0.826
Statin	8.715 (-12.657 – 30.087)	0.067	0.422	23.783 (-35.724 – 83.290)	0.065	0.431
Beta blocker	7.456 (-6.178 – 21.091)	0.089	0.282	20.492 (-17.471 – 58.456)	0.088	0.288
ACE inhibitor or ARB	1.256 (-4.976 – 7.488)	0.033	0.691	-1.871 (-19.229 – 15.486)	-0.018	0.832
Calcium channel blocker	1.664 (-6.529 – 9.857)	0.033	0.689	-2.662 (-25.480 – 20.157)	-0.019	0.818
SGLT2 inhibitor	12.331 (-1.208 – 25.870)	0.148	0.074	36.699 (-0.935 – 74.332)	0.158	0.056

Supplementary Table S2: Uni- and multivariate linear regression analyses including all baseline characteristics as covariates for platelet aggregation in response to ADP 5 µM measured by light transmission aggregometry (LTA) in patients on prasugrel. Abbreviations: ADP, adenosine diphosphate; ACE, angiotensin-converting enzyme; ARB, angiotensin receptor blocker; BMI, body mass index;., B, regression coefficient; CI, confidence interval; CRP, C-reactive protein; TIA, transient ischemic attack.

**Covariates**	**Univariate**			**Multivariate**		
	**B (CI 95 %)**	**Beta**	***p***	**B (CI 95 %)**	**Beta**	***p***
Age	0.265 (0.097 – 0.627)	0.181	**0.048**	0.339 (-0.105 – 0.783)	0.223	0.131
Female Sex	12.718 (3.485 – 21.951)	0.297	**0.008**	20.368 (5.325 – 35.410)	0.499	0.009
BMI	0.568 (-0.278 – 1.414)	0.151	0.185			
Prior mycardial infarction	-1.937 (-10.732 – 6.857)	-0.050	0.662			
Piror stroke or TIA	9.143 (-10.612 – 28.897)	0.104	0.360			
Arterial hypertension	0.246 (-7.734 – 8.226	0.007	0.951			
Hyperlipoproteinemia	-1.9393 (-11.071 – 7.193)	-0.048	0.674			
Peripheral artery disease	-1.455 (-6.338 – 3.427)	-0.048	0.557			
Diabetes mellitus type II	-3.137 (-8.817 – 2.542)	-0.119	0.275			
Smoking	-0.630 (-3.494 – 2.235)	-0.035	0.665			
HAS-BLED Score ≥3	-0.320 (-5.020 – 4.380)	-0.011	0.893			
Serum creatinine	17.379 (3.760 – 30.998)	0.295	**0.013**	-5.183 (-39.948 – 20.582)	-0.059	0.687
Platelet count, G/l	-0.018 (-0.056 – 0.020)	-0.076	0.348			
Hemoglobin	-5.186 (-7.469 - -2.903)	-0.458	**<0.001**	0.780 (-11.359 – 12.919)	0.068	0.898
Hematocrit	-1.893 (-2.674 - -1.111)	-0.482	**<0.001**	-0.265 (-4.526 – 3.995)	-0.068	0.901
High sensitivity CRP	1.761 (0.798 – 2.724)	0.390	**<0.001**	-0.300 (-1.742 – 1.141)	-0.060	0.677
Statin	1.520 (-18.069 – 21.108)	0.012	0.878			
Beta blocker	1.860 (-10.650 – 14.370)	0.024	0.769			
ACE inhibitor or ARB	1.612 (-3.978 – 7.202)	0.046	0. 570			
Calcium channel blocker	0.074 (-7.404 – 7.552)	0.002	0.984			
SGLT2 inhibitor	38.961 (16.402 – 61.520)	0.365	**<0.001**	-1.041 (-32.413 – 30.331)	-0.009	0.947

Supplementary Table S3: Uni- and multivariate linear regression analyses including all baseline characteristics as covariates for platelet aggregation in response to ADP 10 µM measured by light transmission aggregometry (LTA) in patients on prasugrel. Abbreviations: ADP, adenosine diphosphate; ACE, angiotensin-converting enzyme; ARB, angiotensin receptor blocker; BMI, body mass index;., B, regression coefficient; CI, confidence interval; CRP, C-reactive protein; TIA, transient ischemic attack

**Covariates**	**Univariate**			**Multivariate**		
	**B (CI 95 %)**	**Beta**	***p***	**B (CI 95 %)**	**Beta**	***p***
Age	0.287 (0.103 – 0.676)	0.182	**0.047**	0.166 (-0.234 – 0.567)	0.106	0.410
Female Sex	10.959 (1.233 – 20.658)	0.246	**0.028**	10.712 (-2.536 – 23.960)	0.261	0.111
BMI	0.692 (-0.182 – 1.565)	0.177	0.119			
Prior mycardial infarction	-3.790 (-9.868 – 2.289)	-0.100	0.220			
Piror stroke or TIA	1.580 (-12.344 – 15.505)	0.018	0.823			
Arterial hypertension	2.135 (-6.134 – 10.404)	0.058	0.609			
Hyperlipoproteinemia	-2.380 (-8.356 – 3.597)	-0.064	0.433			
Peripheral artery disease	-1.125 (-6.583 – 4.333)	-0.033	0.684			
Diabetes mellitus type II	-3.379 (-9.419 – 2.661)	-0.121	0.269			
Smoking	-0.457 (-3.656 – 2.742)	-0.023	0.778			
HAS-BLED Score ≥3	0.631 (-4.617 – 5.879)	0.019	0.813			
Serum creatinine	13.619 (-0.973 – 28.210)	0.220	0.067			
Platelet count, G/l	-0.023 (-0.066 – 0.019)	-0.087	0.285			
Hemoglobin	-4.695 (-7.137 - -2.253)	-0.400	**<0.001**	5.984 (-5.545 – 17.514)	0.526	0.303
Hematocrit	-1.708 (-2.547 - -0.868)	-0.419	**<0.001**	-2.439 (-6.410 – 1.531)	-630	0.224
High sensitivity CRP	0.552 (-0.050 – 1.155)	0.149	0.072			
Statin	5.026 (-16.851 – 26.903)	0.037	0.651			
Beta blocker	-0.660 (-14.643 – 13.323)	-0.008	0.926			
ACE inhibitor or ARB	3.685 (-2.540 – 9.911)	0.094	0.244			
Calcium channel blocker	2.331 (-6.017 – 10.679)	0.045	0.582			
SGLT2 inhibitor	35.532 (11.729 – 59.336)	0.321	**0.004**	9.122 (-23.670 – 41.913)	0.068	0.580
